# Suicidal behavior, suicidal ideation and patterns among youths in Anywaa zone, Gambella, Southwest Ethiopia: a mixed-methods study

**DOI:** 10.1186/s12888-022-03971-7

**Published:** 2022-06-09

**Authors:** Abreha Addis Gesese, Okani Ojulu Ochan

**Affiliations:** 1 Gambella Teachers’ Education and Health Science College, Department of Clinical Nursing, Gambella, South-West Ethiopia; 2 Addis Ababa University, College of Education and Behavioral Studies, School of Psychology, Addis Ababa, Ethiopia

**Keywords:** Suicidal behavior, Suicidal ideations, Patterns, Youth, Gambella and Southwest Ethiopia

## Abstract

**Background:**

Suicide is a major public health problem and for decades, it has remained one of the leading causes of injury and death worldwide. The objectives of this study were to investigate the prevalence of suicidal behavior, suicidal ideation, and patterns among youth in the Anywaa zone of the Gambella regional state, Southwest Ethiopia.

**Methods and materials:**

A mixed-method study design was used in which a quantitative survey was conducted along with qualitative interviews and FGDs in the Anywaa zone. A total of 136 respondents were included in the survey study from the two woredas. The survey was conducted to assess the prevalence of suicidal behavior and ideations in a sample of preparatory school youth students. A pre-tested and structured questionnaire was used for the descriptive analysis. Qualitative information was also obtained through interviews and focus group discussions to identify the patterns of suicide and to gain more nuanced participants/ survivors’ experiences. Data were analyzed using SPSS version 20, for which descriptive statistics were used. Qualitative data were analyzed using thematic analysis.

**Results:**

Suicidal behaviors and ideation were high among youths in the study area. In this study 62.3% of respondents reported they had heard others talk about their wish to die by suicide, 68 (64.2%) of youth said they had heard many youths claim that “I feel like there is no way out”, 48 (43.3%) reported having seen someone with the signs of planning a suicide such as obtaining a weapon or writing a suicide note. About 68 (64.2%) of participants said, “My family would be better off without me.” The majority of respondents were in the age groups ranging from 26 to 30 years. The results on the patterns of suicide attempts showed that hanging and drug overdose or poisoning were the most common patterns used by both men and women.

**Conclusion:**

The findings indicate that the prevalence of suicide-related behaviors and ideations was high among youths in the Anywaa zone. The results on the patterns of suicide attempts showed that hanging and drug overdose or poisoning were the most common patterns used by both men and women. As a result, we would like to recommend that Government, Non-Governmental Organizations NGOs, and Faith-Based Organizations (FBOs), along with health care providers and counselors should work together by creating awareness, and by establishing Programs that target youths. Meanwhile, early identification and management of suicide risk in youth should be strengthened.

## Background

Globally, suicide is the third leading cause of death among young people aged 10–34 years of age. A nationally representative survey conducted in 2011 found that youth are more vulnerable to suicide. It has been a troubling indication point to a serious problem for youth today [1]. Specifically, suicide was found to be the third leading cause of death among persons aged 10–14 years, second among persons aged 15–34 years, fourth among persons aged 35–44 years, fifth among persons aged 45–54 years, eighth among person 55–64 years, and seventeenth among persons aged 65 years and older [2]. Suicide, as a social issue for families, communities, societies, governments, and policymakers requires psychological and social interventions [3]. Suicide is often a low priority for governments and policy-makers [4].

The highest suicide rate was found in Europe, particularly in Eastern Europe with the lowest prevalence in the Eastern Mediterranean region [5]. In America someone dies by suicide within every 12.95 minutes, and those who attempted suicide were estimated as 1 million with a very high prevalence of suicide among Indigenous and Alaskans [2]. The percentage of people aged 18 or above having suicidal thoughts previously in America was 2.9% among blacks, and 7.9% among people with bicultural intermarriages [6]. Every year in Australia, around 2000 Australians die by suicide (Australian Bureau of Statistics 2011) and in Canada in 2009 alone, there were 11.5 deaths per 100,000 people [7].

The overall annual report of the suicide incidence rate of African countries conducted in 1990 was estimated to be 3.2 per 100,000. In the Global Burden of Disease study of 2010, 49,558 people died by suicide; a median incidence rate of 4.8 per 100,000.There were large variations with higher estimates in South Africa and lower estimates in Zimbabwe, Uganda, Malawi, and Tanzania [8]. From studies conducted in Africa, three were from South Africa which their mean annual estimates was ranged from (10.9 to 32.5 per 100,000 population) [9–11], two from Egypt (0.7 and 2.2 per 100,000 population) [12, 13], two from Senegal mean annual estimated was (0.7 and 3.7 per 100,000 population) (0.7 and 3.7 per 100,000 population) [14, 15], two from Uganda (1.0 and 15.8 per 100,000 population) [16, 17], and two from the United Republic of Tanzania (2.3 and 3.2 per 100,000 population) [18, 19]. In addition, the WHO reports death rates from South Africa [10], and Egypt [12] could be significantly under-reported due to method of data collection.

In Addis Ababa, Ethiopia, suicidal ideation among the adult population was reported as 2.7%, and suicide attempt was 0.9% [20]. A study conducted in Butajira, Southern Ethiopia found that suicide attempt among the adult population was found to be 3.2% [21].

Regarding the patterns used globally, American patterns of most used methods from most to least are, firearms, hanging and suffocation and poisoning /overdose [22, 23]. In African countries, poisoning is the most commonly used method by which people end their lives in rural areas. In Cameroon, for those who died by suicide, primarily women, ingestion of toxic agricultural chemicals was the most common method of suicide (76.6%), followed by hanging (17%) and ingesting non-agricultural toxic chemicals [24]. In South Africa, methods of deaths of men by suicide were, hanging, next to poisoning, followed by firearms, pesticides, falls from height and drowning [25]. According to Ethiopia’s context, hanging and strangulation are the most commonly used methods of suicide (70%), particularly in urban Ethiopia [26]. Poisoning was identified as the second most common method of suicide that was accounted for (19.8%) this evidence was consistent with other Ethiopian studies [20, 21, 27].

Despite a large segment of the population remaining in desperate situations in Ethiopia, limited research has been conducted, with most conducted in developed countries. Few studies, of which a few are qualitative, are conducted in some African countries such as Nigeria, South Africa, Egypt, Cameroon, Uganda, and Tanzania [28, 29]. This study aimed to assess the prevalence of suicide-related behaviors, ideation and patterns among youth using a mixed-method study in the Anywaa Zone, Gambella Southwest Ethiopia.

## Methods and materials

### Study area and period

The study was conducted in Abobo and Gog woreda of the Anywaa zone, Gambella, Southwest Ethiopia between March 1 to 30, 2017, located 776 kms away from Addis Ababa. Based on the 2007 census, the 2017 population projection was 435,999 [30]. The region has 1 general and 4 primary hospitals and 148 health centers and health posts.

### Study design

A mixed methods study among youth in the Anywaa zone was employed given that mixed methods are thought to provide a multidimensional and more substantive understanding of suicide-related ideations, behaviours and patterns. A descriptive survey method wasemployedto quantitatively assess the prevalence of suicide-related behaviors and ideations. The qualitative portion consisting of focus group discussions and in-depth interviews was designed to explore the suicide-related ideations, behaviours and pattern experiences of those who had survived an attempt and parents/relatives of those who died by suicide.

### Sample population

Random sampling generated 136 participants from the two woredas in the quantitative survey study. One hundred six youth and 30 adults from four kebeles and two preparatory schools participated within one of four groups: a. suicide attempt survivor; b. survivor of suicide loss; c. parent/relatives of someone who died by suicide; and d. other youth who were not attending school during school programs.

The study’s purposive sample participants for the qualitative interview portion included 5 parents/relatives of suicide victims and 6 persons/people who attempted suicide. Nineteen randomly selected youths were included in the focus group discussion (FGD). These participants were selected with sex and age considered in the selection process. Informed consent was obtained from all subjects and/or their legal guardian(s).

### Data collection tools and procedures

The quantitative questionnaires included three sections: demographic information, data on suicide-related ideation and behavior prepared in the form of dichotomous variables presented in tables. An adaptation of the Composite International Diagnostic Interview (CIDI) adopted by World Mental Health (WMH) Survey Initiative version of the World Health Organization (WHO) (evaluated in Ethiopia in 2002) was used to assess suicide ideation and attempt. The survey included 27 items of which 12 were dichotomous questions used for the prevalence of suicide-related behavior and ideations [26, 27, 29, 31, 32].

The qualitative one to one interviews followed a semi-structured format with a prepared interview guide consisting of principal and probe questions, both closed and open-ended questions. The guide was designed to encourage conversations in which participants were encouraged to talk freely about each question. Each interview was audio-recorded and transcribed [26, 27, 29, 31, 32]. Demographic characteristics were also collected.

#### Focus group discussions (FGD)

Two focus group discussions (FGD) utilizing a planned and facilitated open-ended discussion guide were held with 19 youth, each group having 9 or 10 participants [26, 27, 29, 31, 32].. The Principal Investigator was assigned as a moderator during the FGDs, in which the discussion points were recorded using audiotapes, and notes were taken during each session lasting between 45 and 60 min. We opted for FGDs because their usefulness in enhancing social interaction different from other qualitative methods, high face validity, and their relevance in providing an opportunity to interview several participants systematically and simultaneously. This method was designed to explore youths’ experiences about the patterns of suicide among women and men. It also enabled the description and understanding of both externally observable behaviors and internal states which permitted the use of all one’s senses and capacities.

### Data quality control and management

Data quality was ensured through careful selection and collection of complete and appropriate data. The clarity and completeness of the data collection formats were checked before the data collection. A 5% sample pretest was performed on a randomly selected resident before the beginning of the study. Thus, the reliability and validity of instruments were evaluated. Data were reviewed and checked for completeness, consistency and relevance prior to being entered and analyzed into SPSS for accuracy.

### Data processing and analysis

#### Quantitative data analysis

The data were cleaned, coded, and entered into Epi data version 3.1and exported to SPSS version 20 for further analysis. Descriptive statistics were performed for socio-demographic data, and patterns of suicide. Frequency distributions and percentages were performed for patterns of suicide and each risk factor. Percentages of risk factors were calculated from the frequencies of high and low number responses.

Interview and focus group discussions were transcribed and initially analyzed by a single researcher using Braun and Clarke’s inductive thematic approach. After reading each interview, any concept coded multiple times was identified as a potential theme. After reading all transcripts, the transcripts were re-examined to be certain no data were missed across the different interviews. The themes were then further refined and reviewed with a co-author and followed the common steps manually based on Braun and Clarke’s thematic approach. Findings are presented in a narrative form.

### Operational definitions and definitions of terms

Suicide: - it is the act of ending one’s life through the means of hanging, poisoning, jumping from height place, shooting, and etc. It can be intentional oneself killing or accident.

Pattern: - is the method or means by which an individual intends to die or die by suicide. E.g. hanging, poisoning and jumping.

Prevalence: -is the number of available cases (both the new and old) in a given year in a specific area.

Youth: - the young people whose ages range from adolescent to early adult stage. The people in the ages ranging from 15 to 34 years and for whom, the majority are dependents with respect to shelter and food.

## Results

### Socio-demographic data of respondents (quantitative part)

Sixty-eight (64.2%) of the respondents were men. The majority of the respondents were aged 18-to-20 years old, followed by 31.1 and 15.1% of youths aged between 21-to-25 and 26-to-30 years old respectively.

The majority of participants were single and 38.7% of participants were married. Sixty five point 1 % of youths were grade 12 students and the remaining were grade 11 students (Table 1).

**Table 1:** Socio-demographic data of the respondent by sex and age among youths in Anywaa zone, Gambella, Southwest Ethiopia: 2017

Variable	Valid	Frequency	Percent
Sex	Male	68	64.2
	Female	38	35.8
Total		**106**	**100.0**
Age in years	15–17	4	3.8
	18–20	59	55.7
	21–25	33	31.1
	26–30	16	15.1
	31–34	4	3.8
Total		**106**	**100.0**
Marital status	Married	41	38.7
	Single	64	60.4
	Divorced	1	.9
Total		106	100.0
Educational Level	Grade 11th	37	34.9
	Grade 12th	69	65.1
Total		**106**	**100.0**

### Prevalence of suicidal behaviors and ideations among the youth

Table 2 presents the prevalence of suicide-related ideation and behavior among youth. Sixty one point 3 % reported that they heard someone saying “life isn’t worth living.” About 68 (64.2%) of participants said, “My family would be better off without me.”More than half of respondents reported that they had heard someone claiming that they will take enough pills to kill themselves and 58.5% of youths identified they wouldn’t be around to deal with suicide.

**Table 2:** Prevalence of suicidal behavior and ideations reported by youth among youths in Anywaa zone, Gambella, Southwest Ethiopia: 2017

Variables	Category	N	Frequency	Percent
Life isn’t worth living	Yes	106	65	61.3
	No	106	41	38.7
My family would be better off without me	Yes	106	68	64.2
	No	106	38	35.8
Next time ‘ll take enough pills to do the job right	Yes	106	56	52.8
	No	106	50	47.2
I won’t be around to deals with that	Yes	106	64	58.5
	No	106	44	41.5
You ‘ll be sorry when I’m gone	Yes	106	76	71.7
	No	106	30	28.3
I won’t be in your way much longer	Yes	106	61	57.5
	No	106	45	42.5
I just can’t deal with everything- life’s too hard	Yes	106	63	59.4
	No	106	43	40.6
Nobody understand me-nobody feel the way I do	Yes	106	74	69.8
	No	106	32	30.2
There is nothing I can do to make it better	Yes	106	61	57.5
	No	106	45	42.5
I’d be better off dead	Yes	106	66	62.3
	No	106	40	37.7
I feel like there is no way out	Yes	106	68	64.2
	No	106	38	35.8
Signs of planning a suicide such as obtaining a weapon or writing a suicide note	Yes	106	48	45.3
	No	106	58	54.7

More than two thirds of participants specified that they have heard many youths say,“You‘ll be sorry when I’m gone.”Nearly 58% of participants reported that they had heard someone say, “I won’t be in your way much longer.” About 60% of youths reported that they have heard someone report they “just can’t deal with everything- life’s too hard”and 74 (69.8%) of youths mentioned that they have heard many people expressed that “Nobody understands” and feels their feelings.

About 57.5% of youths reported that there wassomeonewho said “There is nothing I can do to make it better”, 66(62.3%) respondents reported they had heard some people state their wish to die by suicide, and 68 (64.2%) youths have heard many youths claim that they feel like there is “no way out”. In this study, 48 (43.3%) participants reported that there were signs of planning a suicide such as obtaining a weapon or writing a suicide note (Table 2).

This shows that there was a very high prevalence of suicide-related behavior and ideations in the Anywaa zone among the youth of ages ranging from 15-to-34 years old. The majority of youths of Anywaa zone were extremely aware of not being observed by others to have signs of planning such as obtaining a weapon or writing a suicide note. That means, they use to hide their behaviors not be recognized by others for having suicidal behaviors and ideations.

### Qualitative results

#### Information of persons/people who attempted suicide and its patterns

The majority of respondents who attempted suicide were females and all were agedbetween 20-to-28 years old. The majority of them were married and found in gradesbetween 7-to-10 class. For most in this group, both parents were farmers and uneducated people.

Respondents reported a variety of methods used as seen in the following:

“*At the moment I had small amount of money, I went to the pharmacy to buy drug either to remove the fetus or kill myself. After I bought the drug, I swallowed them all at once. Then, I found myself unconscious and admitted in health center for four days”.* (Interview 1)

The second young man interviewee made the explanation in this way:

*“…….Yes, there was access to the method. The way I tried to kill myself, there was a piece of cover sheet of my small baby, and then, I tried to hang myself in my own house where no one around”*. (Interview 2)

Another young made it like this:

*“I searched and found a rope around our compound. Then, I hang myself in the house while someone was watching at me from outside. Unfortunately, that person came and rescued me from hanging.” (Interview 3)        *

In this study, 67% of youths reported that they had used available materials such as rope and piece of coversheet to hang themselves. 33% of interviewees attempted to die by drug overdose or poisoning. Youths expressed determination to follow through on their plan if their preferred method was not available; looking for alternate lethal means to kill themselves. This was renowned by a respondent as follows:

*“Although I decided to kill myself, there was no lethal means at the moment. I walked in to the forest and prepared a rope. Then, I climbed up on a big tree and hang there. Unfortunately, I fall down and found myself unconscious and body paralyzed.”*(Interview 6), (

**Table 3:** Demographics and patterns of youths who attempted suicide among youths in Anywaa zone, Gambella, Southwest Ethiopia: 2017

Interviewees	Sex	Age	Level of Education	Marital status	Family background	Year of attempt	Patterns of suicide
1st interviewee/ person/people who attempted	F	28	Grade 8th	Married	Farmers	2014	Hanging
2^nd^interviewee/ person/people who attempted	F	20	Grade 10th	Married	police& farmer	2014	drug overdose or poisoning
3rd interviewee/ person/people who attempted	F	21	Grade 8th	Married	Farmers	2015	Hanging
4^th^interviewee/ person/people who attempted	M	25	Grade 9th	Married	Farmers	2013	Hanging
5th interviewee/ person/people who attempted	F	20	Grade 7th	Single	Farmer	2016	drug overdose or poisoning
6th interviewee/ person/people who attempted	M	28	Certificate	Married	Farmers	2011	Hanging

Table 3)

### Information obtained from families/relativesand its patterns

Among the interviewed families/relatives, three were mothers andthe rest were brother and sister. The majority of decedents were females; aged between20-to-30 years old, above grade six in educational status and all were farmers.

Regarding the patterns used, family members were the most likely to know the method by which their loved one died. As a result, families reported as follows;

*“We didn’t observe any sign of suicide from him. He just went to the forest and then, found hang using a rope. We were informed that he was already deceased and we get shocked by the information.* (Interview 1)

She killed herself as:

*“………the means was hanging! She was found hanged herself in her own house in which nobody knows the case.”* (Interview 2)

Their families reported that all those who died suicide found hanging themselves. The majority of the deceased families reported that the majority of deaths were reported out of the house. This was supported by the interviews as follow:

*“She hanged herself on a tree that was found out of home”.* (Interview 4)

*“The means she died by suicide was by hanging on the tree; in the bush”.* (Interview 5) (

**Table 4:** Information of the families /relatives and patterns  among youths in Anywaa zone, Gambella, Southwest Ethiopia: 2017

Interviewees	Relationship	Sex	Age	Educational level	Marital status	Family background	Year of suicide	Patterns
1st interviewee	Brother	M	**24**	10th + 3	Married	Farmers	2014	Hanging
2nd interviewee	Daughter	F	**30**	Grade 6th	Married	Farmers	2013	Hanging
3rd interviewee	Son	M	**25**	Grade 9th	Married	Farmers	2014	Hanging
4th interviewee	Daughter	F	**20**	Grade 10th	Single	Farmers	2015	Hanging
5th interviewee	Sister	F	**22**	Grade 7th	Married	Farmers	2013	Hanging

Table 4)

### Information obtained from the focused group discussion

Two focus group discussions were carried out among purposely selected youths using a semi structured discussion guide with approximately 60% men and 40% women participants, age groups ranged from 16-to-30 years old. Discussion locations were segregated and divided according to their distances (Abobo and Gog woreda/districts).

According to focus group discussion members, two methods were mentioned; hanging and drug over-dose or poisoning.

*“Nowadays, women attemptor die by suicidethrough hanging and drug overdose or poisoning.Whereas,men used only hanging because, there is no means to get firearms”.* (FGD 1)

*Another FGD stated as follows; “when women plan to attempt/die by suicide, they will search for money to buy drug. Sometimes, they pretend themselves to be sick that helps them given money to buy drug. After that, they will swallow all drugs at once either to kill them or make an abortion”*. (FGD 2)

## Discussions

### Prevalence of suicidal behavior and ideations among youths

Our study showed a very high prevalence of suicide-related behavior and ideations among the youths in the Anywaa zone. This study show higher rates than studies in Dangla Ethiopia, where suicide ideation and attempt are reported at 22.5 and 16.2%, respectively [31] or in Butajira, Ethiopia, where the cumulative risk of suicide attempt was 26.3% for major depression, 23.8% for bipolar I disorder [33]. Comparatively, Thailand reported suicidal ideation in the past 12 months as 8.8% [32] and in Japan, the overall 12-month suicidal ideation, plan and attempt rate, was 7.9, 6.8 to 8.9% [34], where10.3% students experienced suicidal ideation in the preceding weeks [35]. In Low and middle-income countries, the 12-month prevalence of suicide attempts was reported as 17.4% among adolescents, which is higher than the prevalence reported in most studies of developed countries [36].

The higher prevalence in the current study might be due to the high-risk factors or drivers of suicide-related ideations and behaviors that contribute to an increased likelihood to die by suicide [37]. It could also be attributed due to the influence of humor expression and depressive emotion of students and parents whereby youths experience poor abstract thinking [35], poor social support, school absenteeism, substance use, and mental illness often associated with suicide among youths [31, 33, 37].

In this study, the majority of youths were extremely aware of not being observed by others to have signs of planning suicide,such as obtaining a weapon or writing a suicide note. They hide their behaviors so as not be recognized by others as experiencing suicide-related behaviors and ideations. Shreds of evidence stated that individuals with suicide-related ideations and behaviors were likely to die soon by suicide. Among several studies, over half of those with suicidal ideation (56.4%) transitioned from suicidal ideation to suicide attemptdriven by high depression score [35]. At the meantime, suicide attempt is one of the most crucial predictors of completed suicide [34]. Previous research suggests that for many patients, the formation of a suicide plan precedes a suicidal act, typically within 1 year of the onset of suicidal ideation [36, 38]. The more detailed and specific the suicide plan, the greater will be the level of risk [39]. A study conducted in Dares Salaam, Tanzania among school-going adolescents showed that 7% of participants had thought about suicide with 6.3% having created a plan to carry out an attempt [37], and in Ethiopia, the overall 12-month prevalence of non-fatal suicidal behaviour, consisting of suicidal ideation, plan and attempt, was7.9% [34].

In this study, suicide-related ideation and behaviors were higher in the ages ranging from 15-to-34 years old, consistent with previous research reporting suicide-related ideation and behaviors being more frequent in younger age groups than in later life [40, 41]. However, in Butajira, younger age was associated significantly with increased non-fatal suicide-related behaviors [34], In Low and middle-income countries suicide attempts are a common problem among adolescents aged 12–18 years [36] however, Yip found that the elderly had the highest suicide rate followed by women aged 20–29 [42].

Our study further revealed that the majority of participants who had attempted suicide or died by suicide were females. This is consistent with similar studies done in Dangila Ethiopia, and similar to results from 40 low-income countries where the majority who died by suicide were women [31, 36]. At this same time our findings contrast to results found in Butajira [33], and Thailand [3]. A study from Butajira, Ethiopia, found that male sex was associated significantly with a suicide attempt that resulted in a fatal outcome, 16.2%, compared with 2.4% in women [34].

### The pattern or method used by youth who attempted suicide

Participants in FGD were asked about the most suicide patterns/methods that the youths used to attempt or who had died by suicide. Respondents identified that hanging and drug overdose or poisoning were the methods used for both those who had made attempts or had died by suicide. Hanging was the most frequent method used by people who had died by suicide. This finding is consistent with previous studies from South Africa where hanging was the most commonly used method, followed by poisoning, and firearms, [25]. In Ethiopia, hanging and strangulation were the most commonly used methods of suicide (70%), particularly in urban Ethiopia among both sexes [26]. In African countries, poisoning was reported as the most commonly used means for people to end their life in rural areas [24].

Relatives/families in this study reported similar results as expressed by these relatives, “Without distinguishing any sign of suicide from him, he went to the forest and hanged himself by a rope.” (Interview 7); “The means was hanging! She hanged herself in her own house where nobody knows”(Interview 8). Once youths decide to kill themselves, they look for and use different methods or patterns of suicide. As the quotes from all groups indicated above, hanging and drug overdose or poisoning were observed as the two major patterns of death by suicide and attempted suicide among the youth in the Anywaa zone. Of the six youth who attempted suicide, 66.7% used hanging while the others used drugs overdose or poisoning. Data obtained from interviews and FGDsimplies that hanging was the most common pattern for both sexes and drugs overdose or poisoning was the most common for women.

Through many heated discussions, participants in the FGD’s came to the common understanding that, men had fewer suicide attempts than women. “Young women are most vulnerable for both to die by suicide and suicide attempt. However, young men will die by suicide when they planned to kill themselves.”

In contrast to the present study, firearm was found the most commonly used method of suicide, followed by hanging or suffocation and poison in America, [22, 23], injury and poisoning [20, 21, 27] in Ethiopia, in other African countries, poisoning was the most commonly used means for people to end their life in rural areas [24]. Reducing suicide demands a standard risk assessments and precautions, [43], age and gender specific approaches [44, 45], development and evaluation of empirically based suicide prevention and treatment protocols [46] among these target age groups, with cooperation, effective policies and program implementations. 

### Strength and limitations of the study

This study tried to address very interesting issues among youths in under-studied settings of Ethiopia. Moreover, this study used a mixed-method approach to dig out the root causes that currently affect youths associated with traditional and community-related issues. Despite the above strength, this study has the following drawbacks. The cross-sectional nature of the study design has constraints on cause-effect relationship of the independent and outcome variables.

## Conclusion and recommendations

### Conclusions

This study concluded that there was a high prevalence of suicide-related behavior and ideations in a community sample of youths in the Anywaa zone, South-West Ethiopia. Given the high prevalence of suicide in this area, no attention has been paid to it. Suicide-related ideation and behaviors were higher in the ages ranging from 15-to-34 years old. Hanging is identified as the primary method to attempt or die by suicide followed by drug overdose or poisoning.

### Recommendations

1. Governmental and non-governmental partners working with and for youth affairs and Faith-Based Organizations (FBOs) could better support by creating awareness on early detection of new suicidal behavior; promoting public awareness of suicidal behavior, its causes, and possibilities for prevention; and increasing the support available to individuals and families targeting specific groups of age 15-to-34 years old.
2. Mental health programming needs to be formed so that Public education initiatives can be responsive to the lives of young people and enhance recognition and understanding of the indicators of suicidal behavior and ideations before the action.
3. Health care providers and counselors (psycho-social workers) should establish programs that target and incorporate the prevention or moderation of behavior-related gaps with the early identification, treatment, and stress management in youth.
4. Adolescent/youth programs (Adolescent and Youth Friendly services) need to be established so that peer-to-peer helping would be carried out among them.
5. Future studies also need to study suicide cases in comparison with other deadly diseases and investigate the position of suicide death among the youth in the Ethiopian context. It is also recommended to study the trends of suicide and critical risk factors that contributes to suicide actions and suicidal behavior in all groups of the population within their diverse settings.

## Data Availability

The data sets used and/or analyzed during the current study are all included in the manuscript.

## Abbreviations

CDC: Center for Disease Control
DSH: Deliberate Self-Harm
FBO: Faith-Based Organizations
FGD: Focus Group Discussion
GBD: Global Burden of Disease
LMICs: Low and Middle Income Countries
NGO: Non-Governmental Organizations
SPSS: Statistical Package for Social Science
WHO: World Health Organizations
